# Development of three-dimensional tissue engineered bone-oral mucosal composite models

**DOI:** 10.1007/s10856-016-5676-7

**Published:** 2016-02-16

**Authors:** Thafar Almela, Ian M. Brook, Keyvan Moharamzadeh

**Affiliations:** School of Clinical Dentistry, University of Sheffield, Claremont Crescent, Sheffield, S10 2TA UK

## Abstract

Tissue engineering of bone and oral mucosa have been extensively studied independently. The aim of this study was to develop and investigate a novel combination of bone and oral mucosa in a single 3D in vitro composite tissue mimicking the natural structure of alveolar bone with an overlying oral mucosa. Rat osteosarcoma (ROS) cells were seeded into a hydroxyapatite/tri-calcium phosphate scaffold and bone constructs were cultured in a spinner bioreactor for 3 months. An engineered oral mucosa was fabricated by air/liquid interface culture of immortalized OKF6/TERET-2 oral keratinocytes on collagen gel-embedded fibroblasts. EOM was incorporated into the engineered bone using a tissue adhesive and further cultured prior to qualitative and quantitative assessments. Presto Blue assay revealed that ROS cells remained vital throughout the experiment. The histological and scanning electron microscope examinations showed that the cells proliferated and densely populated the scaffold construct. Micro computed tomography (micro-CT) scanning revealed an increase in closed porosity and a decrease in open and total porosity at the end of the culture period. Histological examination of bone-oral mucosa model showed a relatively differentiated parakeratinized epithelium, evenly distributed fibroblasts in the connective tissue layer and widely spread ROS cells within the bone scaffold. The feasibility of fabricating a novel bone-oral mucosa model using cell lines is demonstrated. Generating human ‘normal’ cell-based models with further characterization is required to optimize the model for in vitro and in vivo applications.

## Introduction

Composite tissue engineering (TE) constitutes a new avenue particularly in the oral and maxillofacial area where a variety of tissues are closely associated to each other. This proximity often results in the loss of multiple tissue types complicating reconstruction of disfiguring developmental, pathological, or traumatic defects.

Bone loss is a commonly encountered problem that can range from small periodontal defects to more complex, difficult to manage structural defects. Data from National Health and Nutrition Examination Survey (NHNES) showed a high incidence of periodontitis in the US affecting approximately half of the population aged ≥30 years with 8.9 % having sever periodontitis that warrant surgery [1]. A recent multicentre trauma (EURMAT) research which was carried out across several European Oral Surgery centres showed an increasing incidence of maxillofacial trauma resulting in bone loss [2].

Bone loss is conventionally managed by autologous bone grafting. This approach is associated with drawbacks such as co-morbidity of the donor site, limited availability at donor sites, extended surgical time and hospital stays, high incidence of post-operative complications, non-union which may be as high as 69 % [3] and unpredictable bone resorption of up to 50 % of the initial volume [4]. Allografts or xenografts are available, low cost alternatives, but there are ethical/religious issues and concerns with the risk of disease transmission although the overall risk is very low [3]. Osteoconductive synthetic materials are another potential option but their use is limited by the inappropriate mechanical properties, high and unpredictable resorption rates and their lack of osteogenic potential [5].

Soft tissue defects in isolation can be reconstructed by grafting either from oral mucosa or split-thickness skin. However, both are associated with high donor site morbidity. In addition, oral mucosa graft is limited in supply whereas vascularized skin flap contains skin appendages, expresses different keratinization pattern, is easily infected in wet oral environment [6] and heals with scar formation [7]. For composite oral and maxillofacial tissue reconstruction, microvascular free tissue transfer such as osteocutanous radial forearm flap has been widely embraced. Nevertheless, it is associated with significant morbidity including hand ischemia, radius fracture, neurological injuries, and graft failure functionally and aesthetically [8].

TE has offered an opportunity to bypass these shortcomings through many strategies aiming to emulate the normal tissue anatomically and physiologically. Bone engineering with the remarkable advances in biomaterials, scaffold fabrication, stem cell therapy, and molecular biology may provide a potential solution for oro-facial reconstruction [9]. Similarly, an EOM has been developed, characterized, and used for various intra and extra oral applications [10, 11] with the feasibility of a reproducible ex vivo fabrication for treatment of various congenital and acquired intraoral defects [12–14].

Although the progress in TE seems promising, the clinical use is still limited. The experimental applications, on the other hand, provide unique opportunities to investigate the interactions among cells, matrix, biomolecules, and environmental factors that cannot be otherwise studied [15]. In addition, in vitro human constructs may minimize the need for lengthy, costly, and controversial animal studies which can be misleading due to interspecies molecular and physiological differences [16].

While EB and EOM as single separate tissues have received significant attention, only few studies have focused on bone or soft tissue as a part of compound construct [17, 18]. The aim of this study was to develop an in vitro TE composite bone/oral mucosal model mimicking the natural structure of alveolar bone with an overlying oral mucosa. This study has not been previously undertaken and we are the first group to report the development of an in vitro 3D full-thickness osteo-mucosal model containing tissue engineered bone and oral mucosa.

## Materials and methods

### Materials

All materials were purchased from Sigma, UK unless otherwise stated.

### Cell culture conditions

Rat osteosarcoma-derived cell line (ROS) was obtained from liquid nitrogen storage in the School of Clinical Dentistry, University of Sheffield. The cells were cultured in a high glucose complete Dulbecco’s Modified Eagles Medium (CDMEM) supplemented with 10 % v/v foetal bovine serum, 2 mM l-glutamine, 100 U:100 µg ml^−1^ Penicillin–Streptomycin, 625 ng ml^−1^ amphotericin. An immortalized human oral epithelial cell line (OKF6-TERET-2) was kindly provided by Brigham and Women’s Hospital, Harvard Institute of Medicine, USA. The cells were cultured in Green’s medium which consisted of Dulbecco’s Modified Eagle’s medium and Hams F12 medium (Gibco, USA) in a 3:1 ratio supplemented with 10 % foetal bovine serum, 2 mM glutamine, 100 U:100 µg ml^−1^ penicillin–streptomycin, 2.5 µg ml^−1^ amphotericin B, 10^−4^ M adenine, 5 µg ml^−1^ insulin, 2 × 10^−7^ M l^−1^ triiodothyronine, 5 µg ml^−1^ transferrin, 0.4 µg ml^−1^ hydrocortisone, 10 ng ml^−1^ Epidermal growth factor (Invitrogen, USA). Normal oral fibroblasts (NOF) were isolated from keratinized gingival biopsies as previously described [19]. Briefly, biopsies were obtained with written, informed consent from patients attending Charles Clifford Dental Hospital, Sheffield, under ethical approval from National Research Ethics Committee London-Hampstead. The epithelium and dermal layers were enzymatically separated by incubating the tissues in trypsin 1:250 in PBS for 1 h at 4 °C then 2 h at 37 °C. The epithelial layer was scraped and the connective tissue layer of the oral mucosa biopsy was incubated in 0.05 % (w/v) collagenase type I solution (Gibco, USA) in CDMEM at 37 °C overnight. Digested tissue was centrifuge at 200 rpm for 5 min and the pellet was re-suspended in CDMEM and cultured in T-75 cell culture flask.

All cell types were incubated in a humidified atmosphere at 37 °C and 5 % CO_2_/95 % air. The medium was changed 3 times a week until the cells were 90 % confluent when they were sub-cultured while NOFs were passaged up to 2 times.

### Engineered bone model (EB)

9 sterile ceramic discs (3 mm × 20 mm) of Hydroxyapatite/Tricalcium phosphate (HA/TCP) (60 %/40 %) (Ceramisys LTD, UK) with a pore diameter range of 200-450 µm were used as scaffold. Six discs were suspended in a spinner bioreactor (Branstead Stem, UK) to be seeded by cells. 50 µl of ROS cell suspension having a cell density of 2x10^6^ cells were added dropwise to each disc. The cells were allowed to adhere for 2 h and then the cell/scaffold constructs were completely covered with 100 ml of CDMEM and left in the incubator overnight. The spinner was generated after 24 h at a rotation speed of 30 rpm and the medium was changed every 2–3 days for three months. The remaining 3 discs were used as negative control (acellular).

### Engineered oral mucosal model (EOM)

Rat-tail type I collagen was isolated from the tails of Wistar rats as previously described [20]. The extracted collagen was dissolved in 0.1 M acetic acid, freeze dried, and re-dissolved in 0.1 M acetic acid to a stock concentration of 5 mg ml^−1^ and stored at 4 °C. 20 days prior to the end of EB culture, collagen-based EOM was constructed according to the technique described by Dongari-Bagtzoglou [21]. Keeping everything on ice, a solution of DMEM 13.8 mg ml^−1^, FCS 8.5 % (v/v), l-glutamine 2 mM, reconstitution buffer (22 mg ml^−1^ sodium bicarbonate and 20 mM HEPES in 0.062 N NaOH), and 5 mg ml^−1^ rat-tail type I collagen was prepared and neutralized to pH 7.4 by addition of 1 M sodium hydroxide. Finally, cell suspension of primary fibroblasts at a concentration of 1 × 10^6^/model in CDMEM was added to the solution and the resultant fibroblast-containing suspension was distributed into tissue culture inserts (0.4 µm pore size, 30 mm diameter, Millipore) and incubated at 37 °C for 2 h until solidified. Then, 1.5 ml of CDMEM was added inside and outside the insert. The gel was cultured for 3 days until it contracted. Following contraction, 1 × 10^6^ of OKF6/TERT-2 suspended in 50 µl of Green’s medium were seeded on the gel surface and allowed to adhere for 2 h. Subsequently, 2 ml of Green’s medium was gently added into the insert and incubated at 37 °C, 5 % CO_2_ for 3 days. When epithelial cells reached confluency, the culture was raised to the air/liquid interface and fed every other day for 12–14 days.

### Engineered bone-oral mucosa model

Once the culture of the EB and EOM was completed, both were combined using a biocompatible fibrin-based adhesive sealant (ARTISS, Baxter, UK). Briefly, the EBM was retrieved from the spinner flask and placed on a sterile culture plate containing 10 ml of CDMEM. Fibrin was defrosted, the components in pre-filled syringe were mixed, and only a thin layer of the mixed Protein–Thrombin sealer was applied on the dermal side of EOM. Then the latter was immediately attached to the surface of EB and held in the desired position with gentle compression for at least 3 min to ensure ARTISS sets completely and both models were firmly adhered. The bone-oral mucosa construct was further cultured at air/liquid interface for 5 days after which the model was fixed for histological examination.

### Assessments

At the end of each month, the EB models were assessed by; PrestoBlue (PB) vitality assay, histological, and scanning electron microscopy (SEM) examinations. In addition, Micro-computed tomography (micro CT- scan) was used to image the EB at the end of the culture period. Histological examination, SEM, and CT scanning were also carried out for the control discs (acellular).

#### PB assay

PB viability assay (Invitrogen, USA) was used to monitor the cell viability throughout the study. Away from direct light, the old medium in the spinner was aspirated, the seeded discs were washed with PBS and 100 ml of new CDMEM was added. Then, a 1/10th volume of PB reagent was added directly into the spinner and incubated for ≥10 min. Quadruplicate samples of 200 µl were taken and read after incubation for 180 min using spectrophotometric plate reader (Infinite^®^ M200, TECAN, USA). Cell viability was detected with fluorescence (560 nm excitation and 590 nm emission) and the mean values were calculated separately for four independent experiments.

#### Histological examination

EB models were retrieved, fixed in 10 % (v/v) PBS-buffered formalin for 24 h, and decalcified with formic acid for the same period. Specimens were processed overnight using a bench top tissue processor (Shandon Citadel 2000, Thermo Scientific, UK) and embedded in paraffin wax using a Leica EG1160 embedding centre (Leica Microsystems). Then, 10 µm sections were prepared, stained with haematoxylin and eosin (H&E), and examined using inverted microscope equipped with a digital camera (Olympus, Japan).

#### SEM

EB models were rinsed with 5 ml of 0.1 cacodylate buffer, fixed with 3 % of gluteraldehyde for 3 h, and then rinsed again with cacodylate buffer. Osmium tetroxide was added to cover the material surface and left for 2 h. Then, the samples were washed with buffer and dehydrated gradually with the increasing concentration of ethanol solutions (75, 95, and 100 %) for 15 min each. The samples were air dried overnight, then mounted onto 20 mm diameter stubs and their surfaces were sprayed with gold (~20 nm). Cell attachment, adhesion, distribution, and secretion were observed using the SEM (Philips XL-20, USA).

#### Micro-CT

The characteristics of the HA-TCP scaffold and the EB at the end of the experiment were assessed by a high resolution micro-CT scanner (SkyScan 1172; Bruker, Belgium). The sample was covered by polystyrene and positioned horizontally on a brass holder in the centre of the specimen stage. Cross-sectional images were reconstructed and the numerical data was calculated using SkyScan CTvol software.

## Results

### PB assay

PB assay revealed that ROS cells remained vital throughout the experiment with fluorescence emission mean values: 39,136.75 ± 2310.47, 26,140 ± 73.66, 38,567 ± 1681.18, 22,560.25 ± 2561.11 at baseline, 1st, 2nd and 3rd month respectively. The readings showed a slight decline in the cellular metabolic activity after 1 month culture and the end of the 3rd month. PB is considered the fastest resazurin-based assay that gives an accurate reading with short incubation period (10 min to 2 h). In our experiments, however, the optimal readings were obtained after approximately 3 h dye incubation for the colour to turn pink indicating persistent cellular vitality.

### Histological examination

**Fig. 1:**
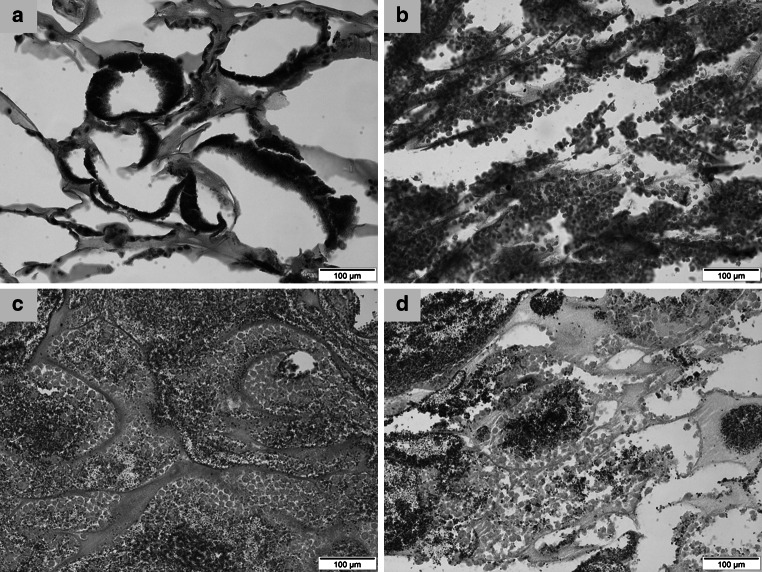
Histological sections of **a** acellular HA/TCP scaffold; **b** EB after one month culture; **c** EB after 2 months culture; and **d** EB at the end of three months culture. H&E staining, original magnification ×20

The histological observation of the EB revealed dense concentration of the cells and even distribution in the 1st month. There was a gradual decrease in density of the cells in the 2nd and 3rd months (Fig. 1a–d).

**Fig. 2:**
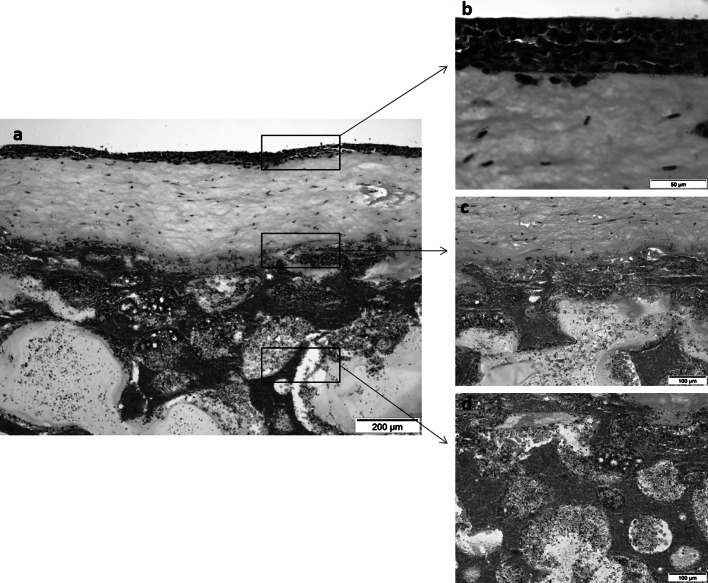
Histological sections of the engineered **a** bone-oral mucosa model; **b** epithelium; **c** connective tissue; and **d** hard tissue layers. H&E staining, original magnification ×10, ×60, ×20, ×20 respectively

Engineered bone-oral mucosa construct (Fig. 2a), on the other hand, showed a relatively differentiated parakeratinized epithelial tissue with 7-9 cell layers of OKF6/TRET-2 (Fig. 2b). Fibroblasts were evenly distributed in the connective tissue layer and bone-oral mucosa interface showed a thin band of fibrin sealant adhering the soft and hard tissues. ROS cells were populated underneath the sealant and scattered around the remaining matrix (Fig. 2c, d).

### SEM examination

**Fig. 3:**
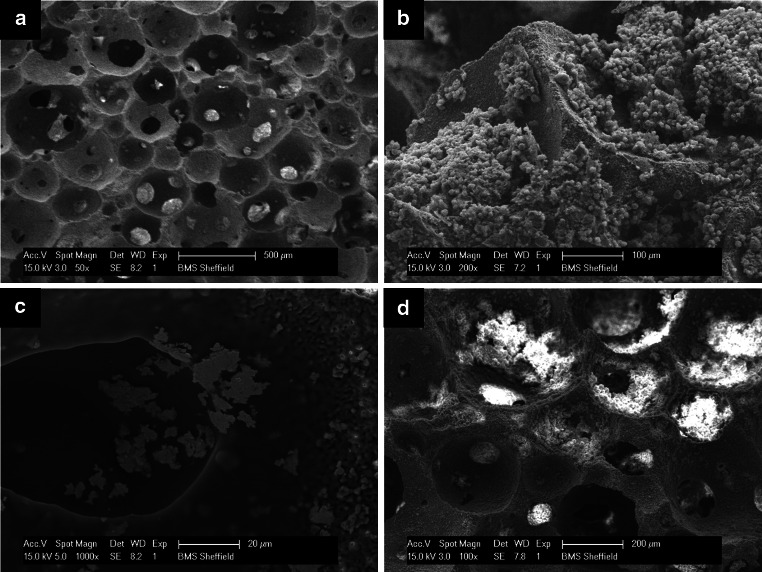
Scanning electron micrographs of **a** acellular HA-TCP scaffold; **b** cell growth within the bone construct in the 1st month; **c** deposition of some calcified nodules in the 2nd month; and **d** mineral deposition and pores closure in the 3rd month

In the 1st month, the surface characteristics of the EB demonstrated the adherent cells proliferated and arranged in dense aggregates or clusters covering the entire scaffold surface and filling the pores. The growing cells had rather a plump, rounded morphology with a thin layer of secreted matrix appearing in few areas (Fig. 3b). In the 2nd and 3rd month, however, the cells revealed a shrunk degenerative appearance at the surface. Some areas were devoid of cells whereas in other areas a calcified matrix and mineral deposition occluding the scaffold pores were observed (Fig. 3c, d).

### Micro CT-scan assessment

A micro-CT was used to analyse the characteristics of acellular HA-TCP scaffold (control) and compare it with the EB at the end of culture (experimental). After three months of dynamic culture in spinner flask, the quantitative 3D analysis of EB revealed a marked increase in closed porosity (volume, percentage, number and surface) and surface density while total and open porosity showed decrease in percentage (Table 1).

**Table 1:** Micro-CT scan analysis of the HA/TCP scaffold and EB at the end of 3 months culture period

	Scaffold	EB
Closed porosity %	0.02	0.06
Volume of closed pores (mm^3^)	0.04	0.17
Bone surface density (1/mm)	15.56	20.16
Surface of closed pores (mm^2^)	9.08	31.65
Open porosity %	67.38	55.33
Total porosity %	67.39	55.36
Volume of open pore space (mm^3^)	422.53	326.81
Total volume of pore space (mm^3^)	422.57	326.98
Connectivity density (1/mm^3^)	390.18	784.66
Number of closed pores	4959.00	11,587.00
Connectivity	244,651.00	463,434.00

## Discussion

In the oral cavity, periosteum and bone are directly attached to the overlying oral mucosa without intervening tissues such as muscle or facia. Success in the fabrication of an in vitro model incorporating hard and soft tissues in one unit will be valuable in investigating the association between these entities instead of examining each tissue individually. In the present work, an in vitro composite construct was developed to mimic oral bone-mucosa structure.

This study demonstrated that improving the quality of culture environment by using spinner bioreactor provided a dynamic microenvironment within the interconnected pores which enhanced nutrient necessary for cells cultivation. This finding is in agreement with many previous studies showing that spinner flask could mitigate the mass transport limitation and promote cells proliferation, differentiation, and expression of osteogenic markers [22, 23]. In addition, the cellular trend observed in ROS/HA-TCP construct was in some way consistent with normal osteoblasts’ growth sequence which consists of three principle periods. First; strong proliferation with ECM formation, second; down- regulation of proliferation accompanied by matrix maturation and up regulation of alkaline phosphatase (ALP) expression, and third; mineralization phase with further decline in proliferation and ALP activity [24]. Similar observations in this study is presumably because ROS cells possess a typical osteoblastic phenotype and responses analogous to those of normal bone cells [25, 26].

The micro-CT analysis which is used to accurately and efficiently segment and characterize the internal structure of bone and bone replacement materials [27, 28] confirmed the data obtained from histological examinations. It may indicate the cell-mediated dissolution processes within the HA-TCP scaffold which resulted in significant increase of percentage, number, and volume of closed pores within the scaffold and increase in the density of the EB.

Although cellular vitality persisted in vitro for a relatively long period (3 months), our qualitative and quantitative investigations revealed a marked decrease in cellularity over time which may indicate necrotic cell death. This may be attributed to the limitation of the spinner bioreactor as it promotes the external mass transport and ECM production at the construct surface, while the dominant nutrient exchange within the construct remains by diffusion [29]. Furthermore, ROS cancer cells have higher proliferation rate and nutritional demand than normal primary osteoblasts which can result in early deprivation from nutrients and cell death which was noticed in this study towards the 2nd and the 3rd months of the culture.

Scaffold size and culture time are other contributing factors that can compromise cell viability. A comprehensive review carried out by Martina and Giuseppe Maria De [30] demonstrated that small size cellular scaffold and/or short in vitro culture period for 14–30 days were predominantly utilized in those studies referred to the advantage of spinner in BE. Meinel et al. [31] cultured human mesenchymal stem cells (MSCs) on 11 mm × 1.5 mm collagen scaffold for 5 weeks. They showed that in spite of porosity and minimal thickness of scaffold, spinner culture did not adequately support mass transport. The penetration depth appeared to be 1 mm or less resulting in bone formation in the exterior and cell death in the centre. Similarly, Zhang et al. [32] compared spinner, perfusion, rotating wall, and biaxial bioreactors for their application in BE and concluded that only the latter achieved high cellularity in large 785 mm^3^ scaffolds.

Long culture periods may further hinder the nutrition by secretion of ECM components such as proteins and proteoglycans which are relatively macromolecules with low diffusion coefficients [33]. Consequently, cells become enclosed by the matrix occluding the open pores as shown in this study.

The morphological features and formation of osteoid-like structures observed in this in vitro model which is based on rat osteosarcoma cell line, are consistent with the generally accepted fact that malignant cells express the differentiated features of the tissue of origin but do not represent the functional properties in terms of cellular products and response which are often species specific [34].

In respect to EOM, and in the process of reproducing and standardizing, normal oral epithelial cell line immortalized by forced expression of telomerase (OKF6/TERT-2) was used instead of normal oral keratinocytes. These cells retain their growth control and differentiation potential in culture as telomerase expression rescues cells from the mechanism of senescence without affecting the major growth behaviour [35]. Underlying connective tissue fibroblasts cultured in this model can proliferate and produce ECM which provide a condition for keratinocytes proliferation and differentiation better than any artificial matrix [36].

One would speculate that an alternative method of generating bone-oral mucosa model would be growing the soft tissue component directly over a piece of bone. However, this may be technically infeasible due to the lack of universal media formulations that would be suitable for different types of cells in a single culture. In addition, this technique may raise the question of how long the cells, particularly in the air lifted epithelium, can survive in the presence of bone that may prevent adequate delivery of medium that is not directly contacted the oral mucosa substitute [18]. Although our composite model revealed that epithelial cells survived for an additional 5 days after final assembly of the full-thickness model in vitro, it must be emphasized that this finding should be interpreted with caution as it does not necessarily represent human primary cells. Further investigations are underway to compare the quality and differentiation status of our newly developed composite tissue model with those of an osteo-mucosal model reconstructed using primary human oral keratinocytes and fibroblasts and normal human alveolar bone-derived osteoblasts.

## Conclusions

The present work indicates that in vitro engineering of bone-oral mucosa model which histologically resembles native human alveolar bone and oral mucosa complex could be established. Combined HA/TCP scaffold seems to be a suitable scaffold for bone engineering although cell vitality can be compromised using ROS cells with high metabolic demand over extended and long culture periods beyond 1 month. The use of fibroblast-populated collagen gel for oral mucosa assembly and employing a biocompatible fibrin-based adhesive to combine the reconstructed soft and hard tissues appear to be successful approaches in TE of a composite osteo-mucosal system. The current findings will ultimately serve as primitive proof of the concept to fabricate an optimised and well- characterised model.
